# Patterns of temporary mechanical circulatory support, escalation, de-escalation and outcomes in cardiogenic shock

**DOI:** 10.3389/fcvm.2026.1720984

**Published:** 2026-07-08

**Authors:** Ibrahim Mortada, Lovkesh Arora, Maria Aguilar Pescozo, Paulino Alvarez, Angelos Soranidis, Alexandros Briasoulis, Ernesto Ruiz Duque

**Affiliations:** 1 Division of Cardiology, Department of Medicine, University of Iowa, Iowa City, IA, United States; 2 Division of Cardiology, Cleveland Clinic, Cleveland, OH; 3 Department of Clinical Therapeutics, National and Kapodistrian University of Athens, Athens, Greece

**Keywords:** cardiogenic shock, ECMO, heart failiure, impella, ventricular failure

## Abstract

**Background:**

Cardiogenic Shock can affect significantly the survival in patients with AMI or ADHF.Mechanical circulatory support is the keystone of treatment beyond standard care to improve end organ perfusion and potentially survival.Hemodynamics progression during support has not been well studied. This retrospective cohort study aims to evaluate the patterns, timingand outcomes of therapeutic circulatory support escalation and de-escalation in patients admitted to a tertiary referral cardiogenic shockcenter.

**Methods:**

Data were collected for the patients over the age of 18 that had implantation of the microaxial flow pump at the University of Iowa HealthCare. Data includes invasive hemodynamics during admission, 24 h and 72 h post intervention along different modalities ofmechanical and pharmacological hemodynamic support in addition to patient history and baseline characteristics.

**Results:**

A total of 110 patients were included in the analysis, 53 patients (48.18%) survived. The average Impella power level was significantly lowerin the survival group (6.21 vs. 6.92, *p*=0.01) despite similar flow rates between groups (3.16 L/min vs. 3.08 L/min, *p*=0.31). 72 h Cardiacpower output was significantly better within survivors (1.15 watts vs. 0.69 watts, p<0.01). Multivariable logistic regression analysis identifiedelevated cardiac power at 72 h post intervention as an independent predictor of survival among patients with GCS during hospitalization(OR 0.09; 95% CI 0.02-0.45; *p*=0.03).

**Conclusion:**

Our data indicate that sustained hemodynamic improvement, particularly in cardiac power output, is associated with survival, independently ofthe absolute changes in Impella flow and power.

## Introduction

Cardiogenic shock (CGS) occurs in 6%–10% of patients with acute myocardial infarction (AMI) (1, 2) and constitutes one of its most notorious complications. Despite evidence-based advances in mechanical circulatory support (MCS) and therapeutic strategies in the management of AMI and CGS, the risk of mortality within 30 days remains elevated reaching in some studies 40%–50% of all patients (3, 4). The microaxial percutaneous ventricular assist device Impella (Abiomed, Danvers, MA) is one of the most widely used MCS devices (5). Impella facilitates left ventricular unloading which reduces myocardial oxygen demand, improves organ perfusion, and increases chances of myocardial recovery (6). Impella is designed to generate antegrade flow of oxygenated blood from the left ventricle into the ascending aorta, reducing left ventricular end-diastolic pressure, lowering myocardial oxygen demand and improving several hemodynamic parameters in patients with cardiogenic shock including increased mean arterial pressure, cardiac output and peripheral tissue perfusion (7). Coronary vessel perfusion is enhanced as well due to the reduction of left ventricular end-diastolic pressure and volume, decreasing left ventricular wall tension and subsequently improving coronary blood flow (8). Despite these hemodynamic benefits, the optimal strategy for mechanical circulatory supported patients with CGS remains uncertain. This retrospective cohort study aims to evaluate the patterns, timing and outcomes of therapeutic circulatory support escalation and de-escalation in patients admitted to a tertiary referral cardiogenic shock center.

## Methods

### Study design

In this retrospective, non-interventional, single center study using Epic electronic health records from the Cardiogenic Shock registry, University of Iowa Hospitals and Clinics (UIHC) (dataset period: January 14, 2018 – October 15, 2024), patients over the age of 18 that had implantation of the Impella microaxial flow pump at the University of Iowa Health Care were selected. The study was submitted and gained approval from the local institutional review board (IRB #202501539).

### Objective

To evaluate the patterns, timing, and characteristics of five distinct temporary circulatory support (TCS) escalation and de-escalation strategies, and their association with mortality, hemodynamic parameters, and laboratory outcomes in patients admitted to a tertiary referral center for cardiogenic shock.

### Study population

Among 182 patients that were supported with MCS, 110 were included in the analysis (Figure 11). Collected patient data included baseline cardiac risk factors, medical history, baseline cardiac testing, use and dosages of inotropes and vasopressors, mechanical circulatory support, and hospital outcomes. The patients were divided into two distinct cohorts, the group consisting of patients who survived until hospital discharge and the in-hospital mortality group.

### Statistical analysis

Continuous variables are reported as median and compared using Wilcoxon rank-sum test. Categorical variables are presented as counts and percentages and compared using chi-square test. Survival was analyzed using a Cox proportional hazards model; covariates included in the multivariable model were selected *a priori* based on their clinical relevance. Kaplan–Meier survival curves were generated to illustrate mortality over time. All significance tests were 2-sided with a significance threshold of *P* < 0.05. Analyses were conducted using STATA, version 18 (StataCorp, College Station, TX).

### Escalation definition

Patients were categorized according to the highest level of TCS escalation during their hospitalization. The no escalation group included patients that received Impella CP as the initial and sole form of TCS. The Impella CP group consisted of patients who were initially supported with an intra-aortic balloon pump (IABP) or with the use of inotropes and were subsequently escalated to Impella CP. The Impella 5.0/5.5 group included patients who were initially supported with an IABP or with the use of Impella CP and were later escalated to Impella 5.0/5.5. The ECMELLA group consisted of patients who were escalated to a combination of venoarterial extracorporeal membrane oxygenation (VA-ECMO) and Impella support. Finally, the VA-ECMO to Impella 5.5 group of patients included anyone who was initially supported with VA-ECMO plus Impella and subsequently decannulated from VA-ECMO while maintaining Impella 5.5 support.

The MCS selection was not protocolized and was determined by the treating cardiogenic shock team according to the patient's clinical status, invasive hemodynamics, severity of shock, response to pharmacologic therapy, and need for escalation or de-escalation of support. For analytic purposes, patients were classified according to the highest level of temporary circulatory support reached during hospitalization.”

## Results

### Baseline characteristics

Baseline characteristics were analyzed for both survivors and non-survivors (Table 1). At baseline level, the mean age was significantly lower among survivors compared to non-survivors (54.51 years old vs. 62.28 years old respectively, *p* < 0.01). Survivors also had lower prevalence of comorbid conditions including type 2 diabetes mellitus (26.4% vs. 49.1% *p* < 0.01), hypertension (26.4% vs. 49.1% *p* < 0.01) and ischemic cardiomyopathy (18.9% vs. 49.1%, *p* < 0.01) (Table 1). Patients with acute decompensated heart failure demonstrated a trend towards higher survival compared to those with AMI, although the difference did not reach statistical significance (66% vs. 49.1%, *p* = 0.07). Out of 52 patients with AMI, 44 had ST elevated myocardial infarction (STEMI) and 8 had non-ST elevation myocardial infarction (NSTEMI). Of the 44 patients with STEMI, 34 (77.2%) underwent percutaneous coronary intervention (PCI) and 34 (77.2%) received revascularization of the culprit lesion. Staging was performed according to the Society for Cardiovascular Angiography and Interventions (SCAI) shock classification system. 17 (15.5%) of patients were classified as stage C, 50 (45.5%) as stage D and 43 (39.1%) as stage E. Survival varied significantly by SCAI stage: 70.6% in stage C, 62% in stage D and 23.3 in stage E (*p* < 0.01). Patients transferred from outside hospitals accounted for 63.6% (70 patients) of our cohort and experienced significantly higher mortality compared to patients who developed shock as inpatients (60.0% vs. 37.5%, *p* = 0.02). No statistically significant difference was found in measured left ventricular ejection fraction (LVEF) between survivors and non-survivors (22.0% vs. 22.5%, *p* = 0.41).

**Table 1:** Baseline characteristics, laboratory and clinical outcomes.

Baseline patient characteristics	Total	%	Admission Survival	%	In Hospital Mortality	%	*p*-value
	N	Median (Q1-Q3)	N	Median (Q1-Q3)	N	Median (Q1-Q3)	
Total	110	100	53	48.18	57	51.82	
Age	60	51–64	58	57–72	63	51–68	0.004
Male	78	70.9	41	77.4	37	64.9	0.12
HTN	61	55.5	24	45.3	37	64.9	0.02
DM	42	38.2	14	26.4	28	49.1	0.01
COPD	9	8.2	5	9.4	4	7	0.66
CKD	29	26.4	14	26.4	15	26.3	0.96
ADHF-CS	58	52.7	34	66	24	49.1	0.07
AMI-CS	52	47.2	19	35.8	33	57.8	
NSTEMI	8	15.4	2	5.7	6	15.8	0.08
STEMI	44	84.6	17	32.1	27	47.4	0.7
None	6	13.6	3	17.65	3	11.11	
PCI	34	77.3	12	70.59	22	81.48	
Angioplasty	4	9.1	2	11.76	2	7.41	
Culprit	34	77.3	13	76.47	21	77.78	0.92
CABG	3	6.8	2	11.76	1	3.7	0.3
CRRT	32	29.1	14	26.4	18	31.6	0.51
SCAI							<0.01
C	17	15.5	12	70.58	5	29.41	
D	50	45.5	31	62	19	38	
E	43	39.1	10	23.25	33	76.74	
Hospitalization							0.02
Transferred	70	63.6	28	40	42	60	
Inpatient	40	36.4	25	62.5	15	37.5	
Laboratory admission							
LDH	783	395–2,121	631	324–1,974	1,198	480–2,478	0.06
Plasma Hb	16	10.8–63	12.5	10.6–83	25	10.8–72.5	0.28
Creatinine	1.67	1.18–2.22	1.49	1.1–2.02	1.8	1.3–2.31	0.04
AST	86	29–524.5	69	22–359	127	42–413	0.07
Lactic Acid	3.2	1.5–3.9	2.3	1.1–7.6	4.45	2.35–5.7	0.0,002
Hb	11.78	9.7–13.9	12.0	10.7–14.3	11.1	9.4–13.9	0.007
WBC	12.4	8.8–17.4	10.6	7.9–15.3	15.45	10.5–19.7	0.006
LVEF	20	15–25	20	15–25	20	15–25	0.71
ACT eval	37	33.63	30	81.08	7	18.91	<0.01
None	73	66.36	23	31.5	50	68.49	
LVAD	12	32.43	10	83.33	2	16.66	
Transplant	16	43.24	16	100	0	0	
Declined	7	18.91	2	28.57	5	71.42	
Recovered	25	22.72	25	100	0	0	

COPD, chronic obstructive pulmonary disease; CKD, chronic kidney disease; ADHF-CS, acute decompensated heart failure-cardiogenic shock; AMI-CS, acute myocardial Infarction-cardiogenic shock; NSTEMI, non-ST elevation myocardial infarction; STEMI, ST elevation myocardial infarction. Percutaneous Coronary Intervention; CRRT, continuous renal replacement therapy; SCAI, society for cardiovascular angiography & interventions; LVEF, left ventricular ejection fraction; ACT Evaluation, advanced cardiac therapies evaluation; LVAD, left ventricular assist device.

### Advanced cardiac therapy evaluation and in-hospital survival

37 patients (33.6%) underwent evaluation for advanced cardiac therapies. Among the 37 patients evaluated, 12 (32.4%) received a left ventricular assist device (LVAD) with a survival rate of 83.33% and 16 (43.24%) underwent heart transplantation with 100% survival.

Additionally, out of 110 patients, 25 (22.72%) were weaned off from mechanical circulatory support due to myocardial recovery and survived to hospital discharge.

Among 110 patients included in our study, 53 patients (48.18%) survived until hospital discharge while 57 patients (51.82%) passed away during their hospital stay.

### Hemodynamics, laboratory findings, impella settings, inotrope and vasopressor requirements prior the intervention

Hemodynamic parameters obtained within 24 h prior to Impella implantation showed significant differences between survivors and non-survivors (Table 2). Patients in the survival group had a lower mean right atrial (RA) pressure (17 mmHg vs. 20 mmHg, *p* = 0.008), higher venous oxygen saturation (SvO2) (57% vs. 43%, *p* = 0.0003) and higher cardiac power output (0.67 watts vs. 0.50 watts, *p* = 0.002). On admission, the survival group had lower lactic acid levels (2.3 mmol/L vs. 4.45 mmol/L, *p* = 0.0002), higher hemoglobin (Hb) (12.0 g/dL l vs. 11.1 g/dL, *p* = 0.007), and lower white blood cells (WBC) count (10.6 k/mm3 vs. 15.45 k/mm3, *p* < 0.01). The average Impella power level required was significantly lower in the survival group compared to non-survivors (6.0 vs. 7.0, *p* = 0.006) despite similar flow rates between groups (3.2 L/min vs. 3.4 L/min, *p* = 0.78). Regarding inotropic and vasopressor support at admission, only norepinephrine dosing was found to significantly differ between survivors and non-survivors (0.13 mcg/kg/min vs. 0.22 mcg/kg/min, *p* = 0.04), with dobutamine, milrinone, epinephrine and vasopressin exhibiting no significant difference.

**Table 2:** Comparison outcomes admission of hemodynamics, pharmacological and mechanical circulatory support.

Admission Hemodynamic	Total	SD	Admission Survival	SD	In Hospital Mortality	SD	*p*-value
	N	Median (Q1-Q3)	N	Median (Q1-Q3)	N	Median (Q1-Q3)	
Systolic Blood Pressure	95	86–110	95	86–111	94	84–106	0.39
Diastolic Blood Pressure	65	55–74	67	57–75	62	51–71	0.14
Mean Arterial Pressure	73	67–82	76	69–83	71	64–80	0.07
Pulse Pressure	29	20–41	29	12–20	30	21–42	0.65
Right Atrium Mean Pressure	18	14–22	17	43–60	20	15–24	0.008
Pulmonary Arterial Systolic Pressure	51	43–60	51	43–60	50	43–60	0.52
Pulmonary Arterial Diastolic Pressure	30	24–32	29	24–32	30	23–34	0.48
Pulmonary Arterial Mean Pressure	38	32–43	38	32–43	37	32–43	0.83
Pulmonary Capillary Wedge Pressure	27	23–30	26	22–30	28	23–32	0.21
Pulmonary Vascular Resistance	2.68	1.56–3.53	2.7	1.4–3.88	2.5	1.56–3.53	0.91
Mixed venous oxygen saturation (%)	49.8	41–61.9	57	48.4–66.65	43	37–53	0.0003
Cardiac Output Fick	3.61	2.76–4.31	3.9	2.97–5.11	3.3	2.56–4.0	0.01
Cardiac Index Fick	1.78	1.39–2.06	1.9	1.57–2.36	1.7	1.29–1.93	0.008
Cardiac Output Thermodilution	3.62	2.99–4.49	3.9	3.23–5.39	3.3	2.86–4.07	0.01
Cardiac Index Thermodilution	1.76	1.46–2.22	1.9	1.47–2.72	1.7	1.41–1.98	0.02
Systemic Vascular Resistance	1,259	933–1,690	1,259	1,010–1,690	1,243	898–1,747	0.89
Cardiac Power Output	0.57	0.43–0.77	0.67	0.53–0.90	0.5	0.4–0.69	0.002
Inotrope							
Dobutamine	5	2.5–5	5	**2.5–5**	3	**2.5–5**	0.24
Milrinone	0.375	0.25–0.5	**0.375**	**0.25–0.5**	**0.375**	0.24–0.5	0.84
Norepinephrine	0.2	0.07–0.3	0.13	0.05–0.2	0.22	0.11–0.4	0.04
Epinephrine	0.05	0.03–0.15	0.05	0.03–0.15	0.05	0.03–0.15	0.92
Vasopressin	0.04	0.04–0.04	0.04	0.04–0.04	0.04	0.04–0.04	0.54
Mechanical Circulatory Support							
Impella Power	7	6	6	5	7	6	0.006
Impella Flow	3.2	2.8	3.2	2.8	3.4	2.8	0.78

SVR, systemic vascular resistance (dyn·s·cm⁻⁵); PVR, pulmonary vascular resistance (Woods Units).

### Hemodynamics, LDH levels and impella settings 24 h post intervention

Hemodynamic data collected 24 h post Impella implantation demonstrated significantly greater improvement in the survival group (Table 3). Compared with non-survivors, these patients had higher SVO2 (57% vs. 43%, *p* = 0.0003), cardiac output (CO) (3.9 L/min vs. 3.31 L/min, *p* = 0.01), cardiac index (CI) (1.9 L/kg/min vs. 1.7 L/kg/min, *p* = 0.01), and cardiac power output (0.67 watts vs. 0.50 watts, *p* = 0.002). The median Impella power level in both groups had no significant difference (6 vs. 7, *p* = 0.26). However, the survival group demonstrated significantly higher Impella flow (3.3 L/min vs. 3.0 L/min, *p* = 0.04). Furthermore, survivors required lower doses of norepinephrine (0.03mcg/kg/min vs. 0.16 mcg/kg/min, *p*-0.04) and epinephrine (0.05 mcg/kg/min vs. 0.06 mcg/kg/min, *p* = 0.04) when compared to non-survivors.

**Table 3:** Comparison outcomes 24 h post intervention of hemodynamics, pharmacological and mechanical circulatory support.

24 h Hemodynamic post Intervention	Total	N	Admission Survival	N	In Hospital Mortality	N	*p*-value
	N	Median (Q1-Q3)	N	Median (Q1-Q3)	N	Median (Q1-Q3)	
Right Atrium Mean Pressure	12	8–14	10	7–13	12	9–14	0.06
Pulmonary Arterial Systolic Pressure)	44	36–54	46	33–55	42	37–50	0.55
Pulmonary Arterial Diastolic Pressure	21	17–25	21	17–27	21	18–25	0.91
Pulmonary Arterial Mean Pressure	29	24–35	30	25–38	29	24–33	0.31
Pulmonary Capillary Wedge Pressure	20	17–25	21	17–25	20	18–25	0.88
Pulmonary Vascular Resistance	1.97	1.34–2.54	1.97	1.22–2.63	1.97	1.53–2.54	0.58
Mixed venous oxygen saturation (%)	59	48–67	61.2	55–68	54.7	43–64	0.001
Cardiac Output Thermodilution	4.52	3.6–5.3	4.7	4–5.5	4.1	3.25.12	0.03
Cardiac Index Thermodilution	2.29	1.73–2.63	2.37	2.1–2.74	2.12	1.5–2.4	0.01
Systemic Vascular Resistance	1333	1,083–1,667	1,303	1,057–1,503	1,348	1,115–1,728	0.3
Systolic Blood Pressure	93	82–104	95	85–105	89	74–97	0.02
Diastolic Blood Pressure	64	58–70	66	60–72	63	55–70	0.03
Mean Blood Pressure	84.5	78–95	87	82–96	82.6	72–90	0.02
Pulse Pressure	28	17–39	30	20–40	26	9.5–37	0.09
Cardiac Power Output	0.81	0.53–0.97	0.86	0.67–1.06	0.71	0.42–0.92	0.02
Plasma Hemoglobin							
Lactate dehydrogenase	743	362–1,835	500	305–1,794	1,154	514–2,604	0.01
Inotrope							
Dobutamine	5	2.5–5	5	2.5–5	5	2.5–5	0.95
Milrinone	0.375	0.25–0.375	0.375	0.31–0.43	0.312	0.25–0.375	0.35
Norepinephrine	0.1	0.03–0.3	0.03	0.02–0.07	0.16	0.06–0.4	0.02
Epinephrine	0.05	0.02–0.1	0.05	0.04–0.06	0.06	0.02–0.12	0.04
Mechanical Circulatory Support							
Impella Power (Watts)	6	5–7	6	5–7	7	5–7	0.25
Impella Flow (L/min)	3.1	2.6–3.6	3.3	2.7–3.9	3	2.45–3.5	0.03

SVR, systemic vascular resistance (dyn·s·cm⁻⁵); PVR, pulmonary vascular resistance (Woods Units).

### Hemodynamics, lactic acid levels, hemoglobin and impella settings 72 h post intervention

At 72 h post Impella implantation the survival group continued to demonstrate statistically significant hemodynamic improvements compared to the non-survival group of patients (Table 4). These patients exhibited higher SVO2 (61% vs. 55%, *p* = 0.01), cardiac output (5.63 L/min vs. 4.39 L/min, *p* = 0.0002), cardiac index (2.83 L/kg/min vs. 2.10 L/kg/min, *p* = 0.0001), and cardiac power output (1.14 watts vs. 0.77 watts, *p* < 0.01). The survival group also had a significantly lower mean level of lactic acid (1.0 mmol/L vs. 2.1 mmol/L, *p* < 0.01). Plasma free hemoglobin levels were not significantly reduced in the survivor group (from 31 g/dL to 41 g/dL, *p* = 0.8). There were no significant differences in Impella power level between the groups (6.0 L/min vs. 5.0 L/min, *p* = 0.59) or Impella flow (3.3 L/min vs. 2.8 L/min, *p* = 0.11).

**Table 4:** Comparison outcomes 72 h post intervention of hemodynamics, lactic acid, length of stay, complications, VA ECMO mean flow levels.

72 h Hemodynamic Post Intervention	Total	SD	Admission Survival	SD	In Hospital Mortality	SD	*p*-value
	N	Median (Q1-Q3)	N	Median (Q1-Q3)	N	Median (Q1-Q3)	
Right Atrial Mean Pressure	10.5	7.0–15	9	6.0–12	13	8.0–17	0.001
Pulmonary Arterial Systolic Pressure	40	34–48	39	33–46	40	35–51	0.31
Pulmonary Arterial Diastolic Pressure	19	15–24	18	13–23	21	17–28	0.01
Pulmonary Arterial Mean Pressure	28	23–33	25	21–31	28	24–34	0.11
Pulmonary Capillary Wedge Pressure	19	15–24	17	13–23	20	17–28	0.008
Pulmonary Vascular Resistance	1.53	1.05–2.23	1.42	0.99–1.95	1.71	1.2–2.53	0.08
Mixed venous oxygen saturation (%)	59	45.6–66.3	61	54–67	55	44–62.8	0.01
Cardiac Output Thermodilution	5	4.2–6.12	5.63	4.64–6.85	4.39	3.14–5.45	0.0002
Cardiac Index Thermodilution	2.6	2.07–2.98	2.83	2.4–3.19	2.1	1.69–2.6	0.0001
Systolic Blood Pressure	98.5	82–108	104	95–114	89	73–101	<0.01
Diastolic Blood Pressure	62	54–70	64	60–73	58	49–65	0.001
Mean Blood Pressure	87.8	73–96	93	84–73	80	64–90	<0.01
Pulse Pressure	32	19–44	38	25–50	28	8.0–40	0.002
Cardiac Power Output	0.92	0.6–1.23	1.14	0.87–1.53	0.77	0.34–0.93	<0.01
Lactic Acid	1.4	0.8–3.0	1	0.7–1.7	2.1	1.3–8.3	<0.01
MCS							
Impella Power	6	3.0–7.0	6	3.0–7	5	4.0–7.0	0.59
Impella Flow	3	2.2–4.79	3.3	2.25–3.9	2.8	1.69–3.3	0.11
Length of Stay	15	5.0–42	33	17–58	5	2.0–11.5	<0.01
Bleeding	23	20.9	11	20.75	12	21.05	0.96
Limb Ischemia	6	5.54	3	5.66	3	5.26	0.92
Peak Plasma Hemoglobin	38	13.6–114	31	13.7–100	41	13.05–118	0.8
VA ECMO	15		6	11.3	9	15.8	0.49
VA ECMO flow	4.35	3.75–4.79	4	3.1–4.3	4.78	2.0–4.85	0.07

MCS, mechanical circulatory support; LDH, lactate dehydrogenase; VA ECMO, veno-arterial.

extracorporeal membrane oxygenation.

### Predictors of survival

**Figure 1:**
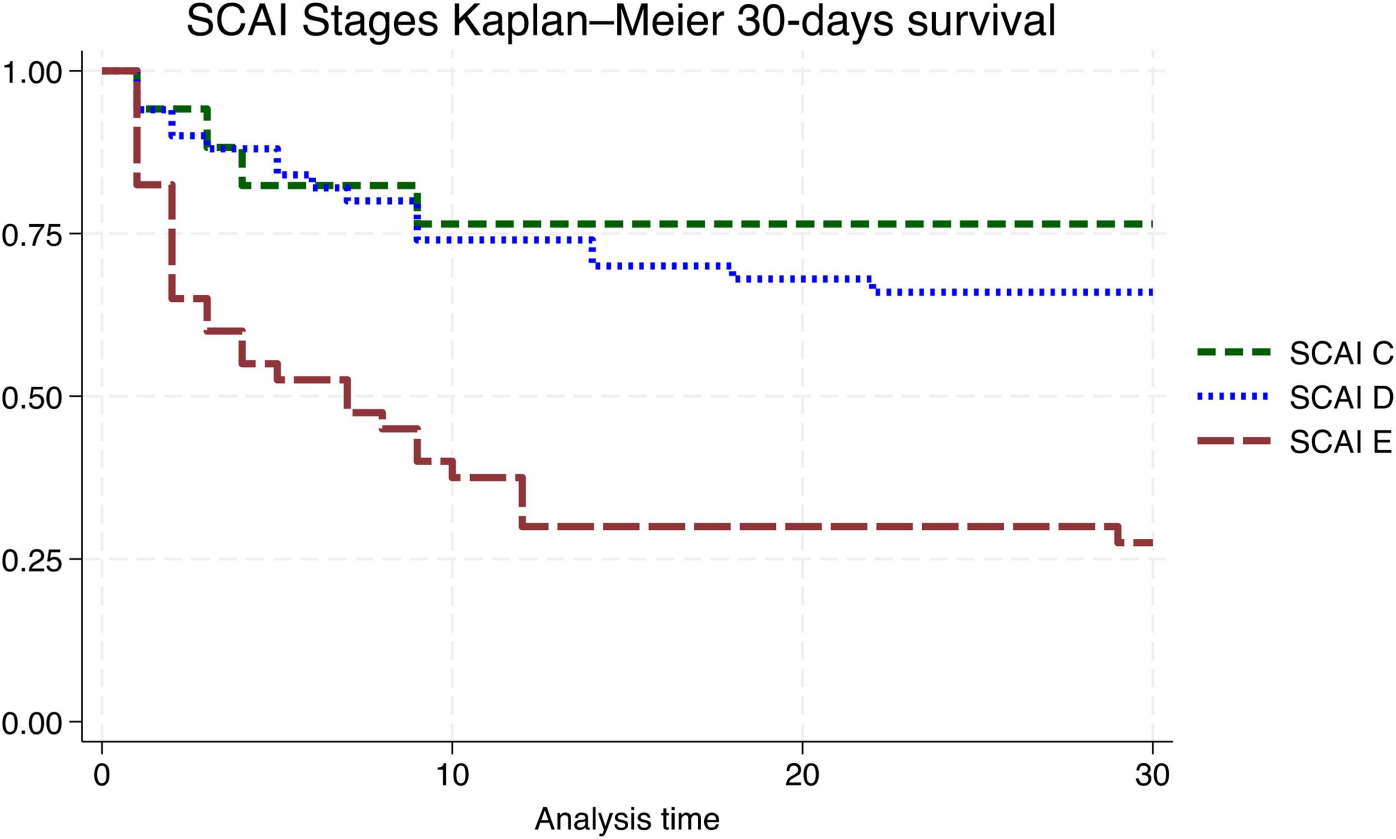
SCAI, society for cardiovascular angiography & interventions kaplan meier 30-days survival. Kaplan Meier 30-days survival cox proportional hazard ratio model 2.34 (95% CI 1.50–3.64) *p* < 0.01.

Univariate analysis demonstrated that patients classified as SCAI stage E had nearly an eightfold increased risk of mortality compared with those in stages C or D [Odds Ratio [OR] 7.91; 95% Confidence Interval [CI], 2.24–27.93; *p* < 0.01]. Additionally, transfer from another hospital was associated with more than a twofold increase in mortality risk (OR, 2.5; 95% CI 1.12–5.55; *p* < 0.01) compared to patients that did not. Elevated serum lactate was associated with a 29% increase in mortality risk. Conversely, higher cardiac power output at 72 h post-intervention had a strongly protective effect, decreasing the risk of in-hospital mortality (OR, 0.08; 95% CI 0.02–0.27; *p* < 0.01). Furthermore, multivariable logistic regression analysis identified elevated cardiac power output at 72 h post intervention as an independent predictor of survival among patients with GCS during hospitalization (OR 0.09; 95% CI 0.02–0.45; *p* = 0.03) (Table 5). Kaplan Meier analysis revealed that the SCAI staged C cohort had the highest 30-days survival, followed by SCAI staged D, and then SCAI E, with a cox proportional hazards model showing increased mortality risk across stages [Hazard Ratio (HR) 2.34; 95% CI 1.50–3.64; *p* < 0.01] (Figure 1).

**Table 5:** Logistic regression analysis cardiogenic shock in-hospital mortality.

Log regression	Univariate Analysis		Multivariate Analysis	
SCAI	OR (95% confidence interval)	*P* Value	OR (95% confidence interval)	*P* Value
D	1.47 (0.44–4.83)	0.52	1.65 (0.38–7.02)	0.49
E	7.91 (2.24–27.93)	<0.01	3.11 (0.61–15.84)	0.17
Transfer vs. Inpatient	2.5 (1.12–5.55)	0.02	1.40 (0.46–4.21)	0.54
Cardiac Power Output 72 h	0.08 (0.02–0.27)	<0.01	0.09 (0.02–0.45)	0.003
Lactic Acid 72 h	1.29 (1.08–1.54)	<0.01	1.14 (0.96–1.36)	0.11

SCAI, society for cardiovascular angiography & interventions.

### Mechanical circulatory support and escalation strategy

We compared Impella power settings, flow rates, cardiac power output and mechanical circulatory support (MCS) escalation patterns at three key time points: Admission, 24- and 72-hours post-implantation.

**Figure 2:**
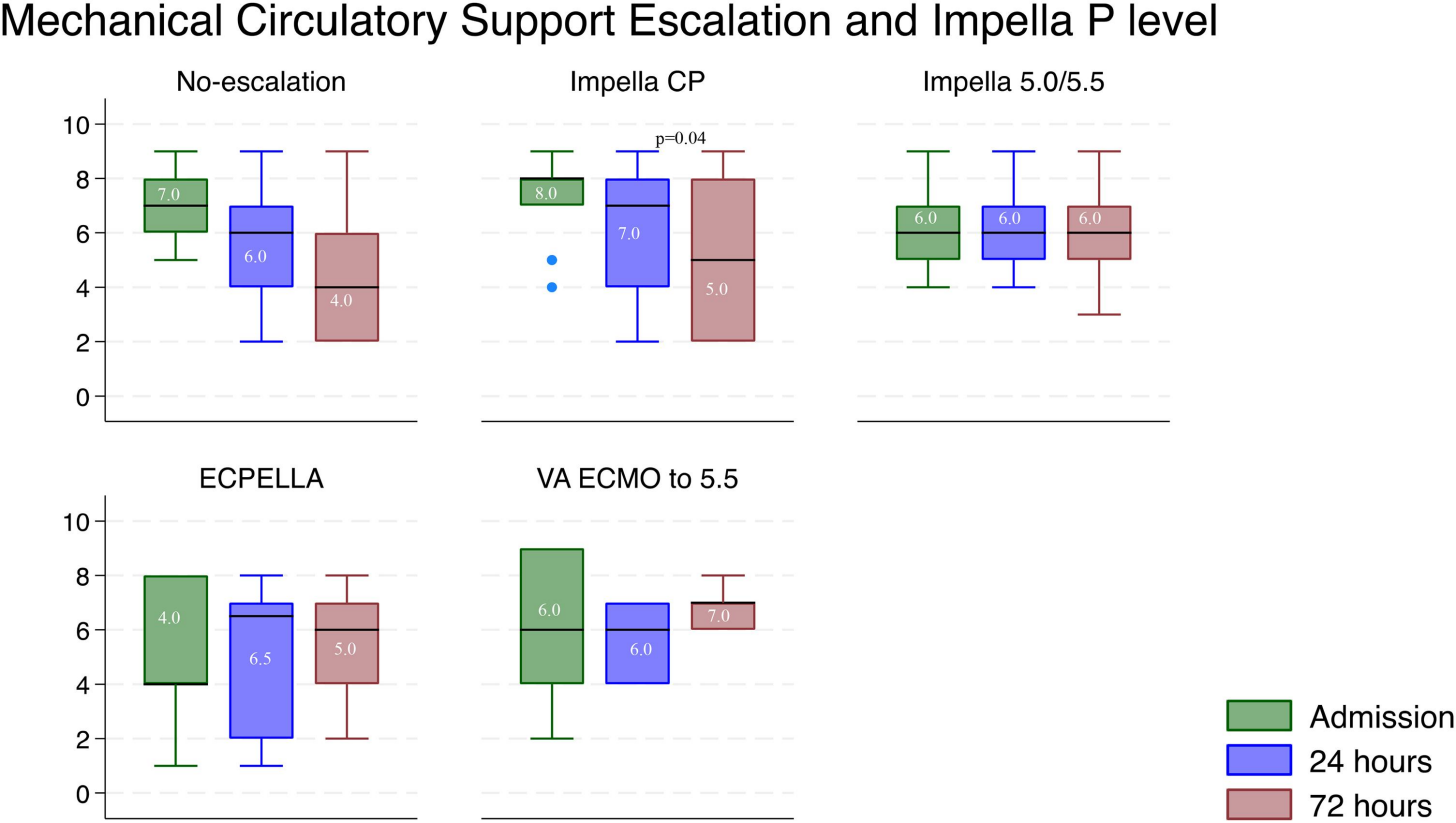
Comparison of the impella power, and MCS escalation at different times of the hospitalization (admission, 24 h and 72 h).

In the no escalation group, Impella power levels declined significantly over time, from admission to 72 h post-implantation (Figure 2). Within this cohort, 8% underwent orthotopic heart transplantation (OHT), 36% experienced myocardial recovery and 56% died during the hospitalization.

Similarly, in the Impella CP cohort, Impella power levels decreased significantly across the same time intervals (Figure 2). In this group, 5% received OHT, 24% had myocardial recovery, and 71% died during their hospital stay.

In contrast, in the Impella 5.0/5.5, ECMELLA and VA ECMO to Impella 5.5 groups, there was no statistically significant change in the Impella power level across the timepoints of admission, 24-hours and 72-hours post intervention (Figure 2). The Impella 5.0/5.5 subgroup demonstrated the highest use of advanced therapies with 29% receiving OHT, 32% receiving a LVAD and 31% passing away during their hospitalization. In the ECMELLA cohort of patients, 0% received OHT or LVAD, 10% had myocardial recovery, and 90% died during the current admission. Regarding the VA ECMO to Impella 5.5 group 40% received OHT, 20% received LVAD, 20% had myocardial recovery, and 0% died during the admission.

**Figure 3:**
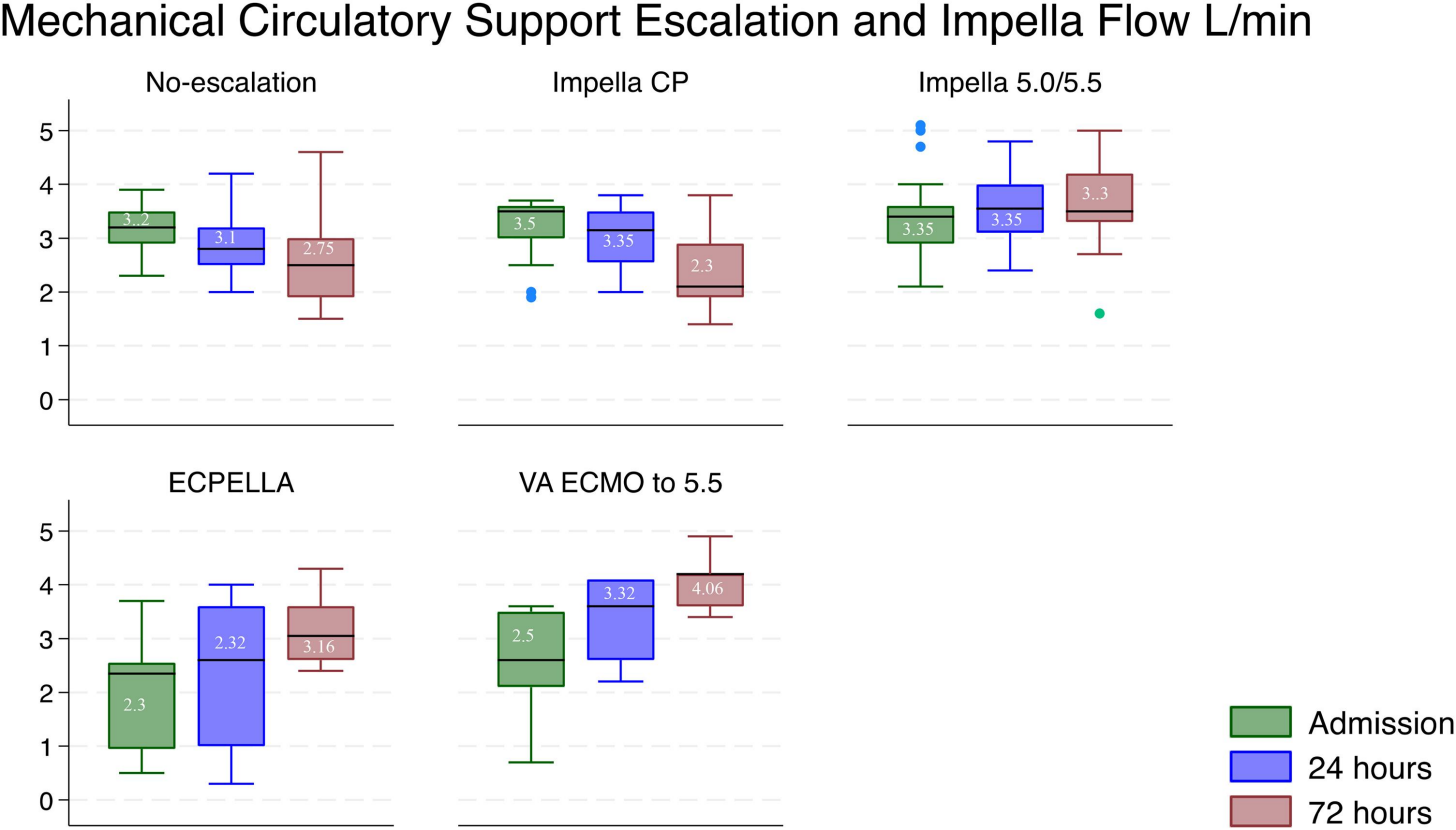
Comparison of the impella flow, and MCS escalation at different times of the hospitalization (admission, 24 h and 72 h).

Regarding Impella flow rates, the no escalation group and patients with escalation to Impella CP cohort had a decrease of flow rate from admission to 72 h post intervention. The group of VA ECMO to Impella 5.5 cohort had an improvement of the flow despite stable Impella P level (Figure 3).

**Figure 4:**
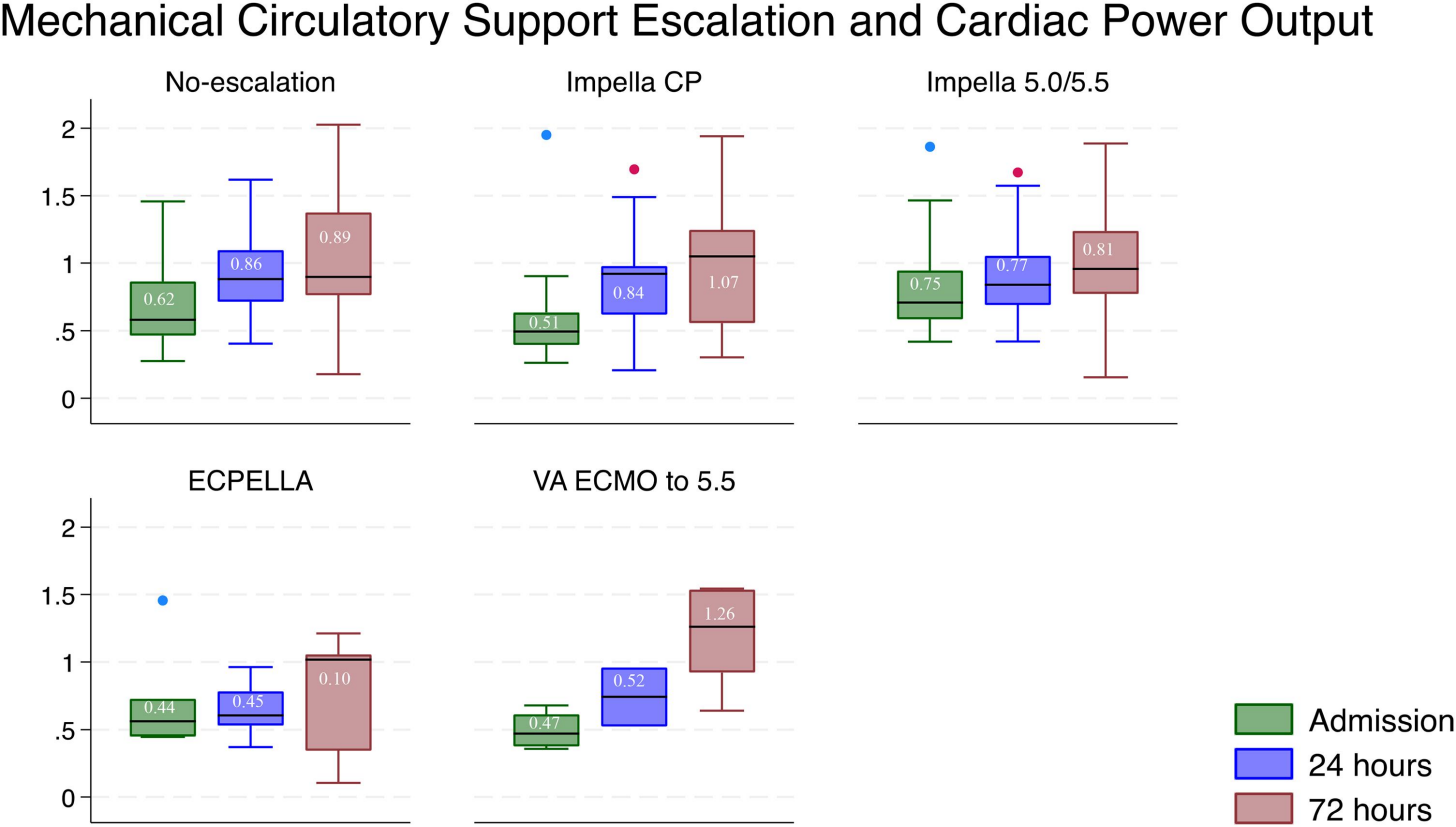
Comparison of the cardiac power output, and MCS escalation at different times of the hospitalization (admission, 24 h and 72 h).

Cardiac power output demonstrated a significant stepwise increase in the no escalation group, Impella CP group, Impella 5.5 and VA ECMO to Impella 5.5 cohort with a significant deterioration of the CPO only on ECPELLA group. (Figure 4).

## Discussion

In this single-center retrospective study, we collected several novel hemodynamic and clinical variables during the first 72 h of admission for patients with cardiogenic shock. Our findings yield important insights into the use of MCS and its impact on clinical outcomes, highlighting distinct patterns and their hemodynamic correlates.

Baseline evaluation revealed that patients who survived hospital discharge were significantly younger, had lower prevalence of comorbid conditions and had a more favorable SCAI staging. Additionally, patients who transferred from another hospital were found to have an increased mortality risk, potentially due to the severity of their condition that prompted their transfer to our facilities.

**Figure 5:**
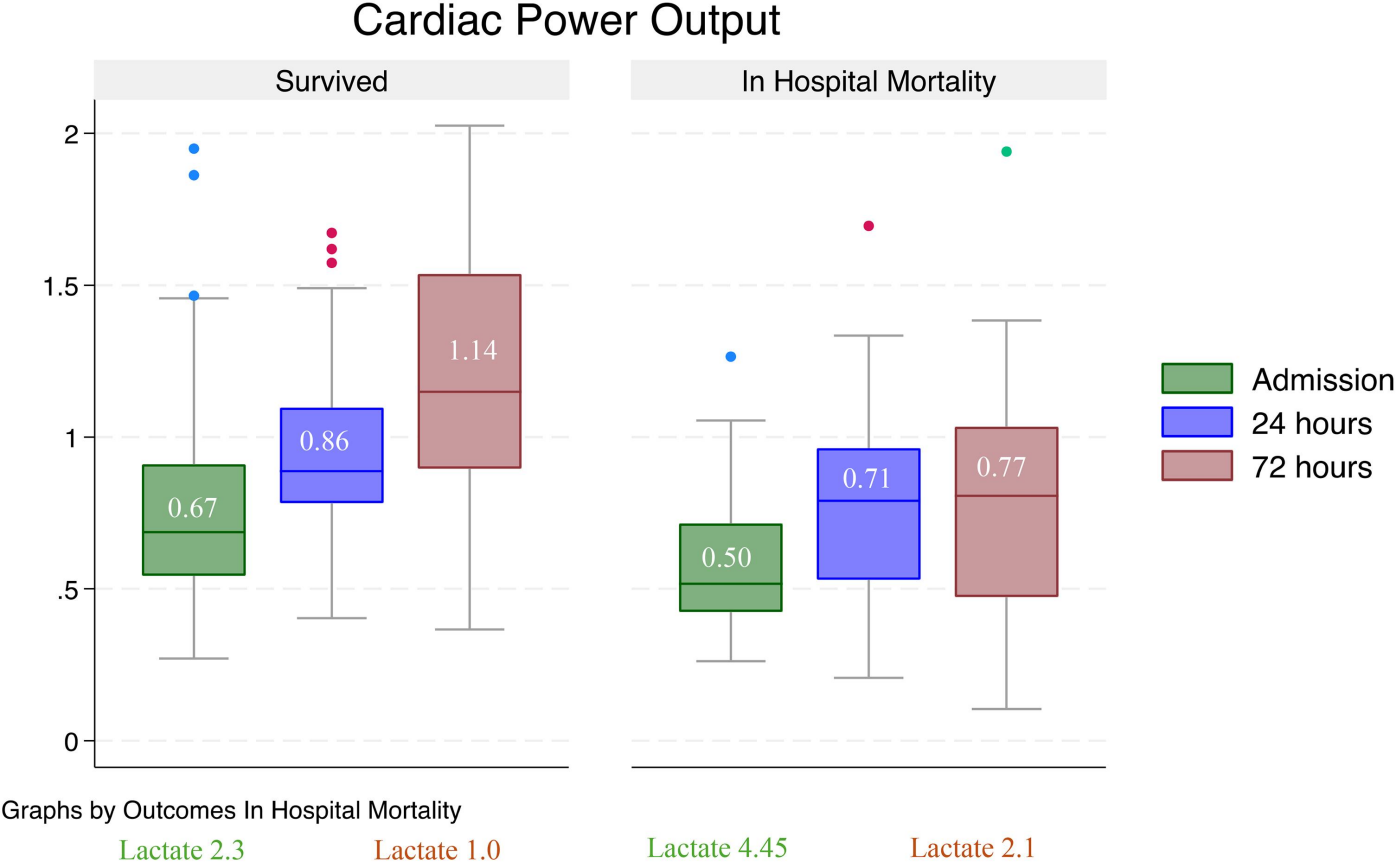
Cardiac Power Output (CPO) during admission, 24 hours, and 72 hours post intervention and Outcomes. The cardiac power output improved in the survived group across whole 24 h and 72 h post intervention with an improvement of lactate. Among the patients that did not survive there was significant improvement within the first 24 h that did not persist by the 72 h post intervention. Similarly, there was a not normalization of the lactate by the 72 h.

**Figure 6:**
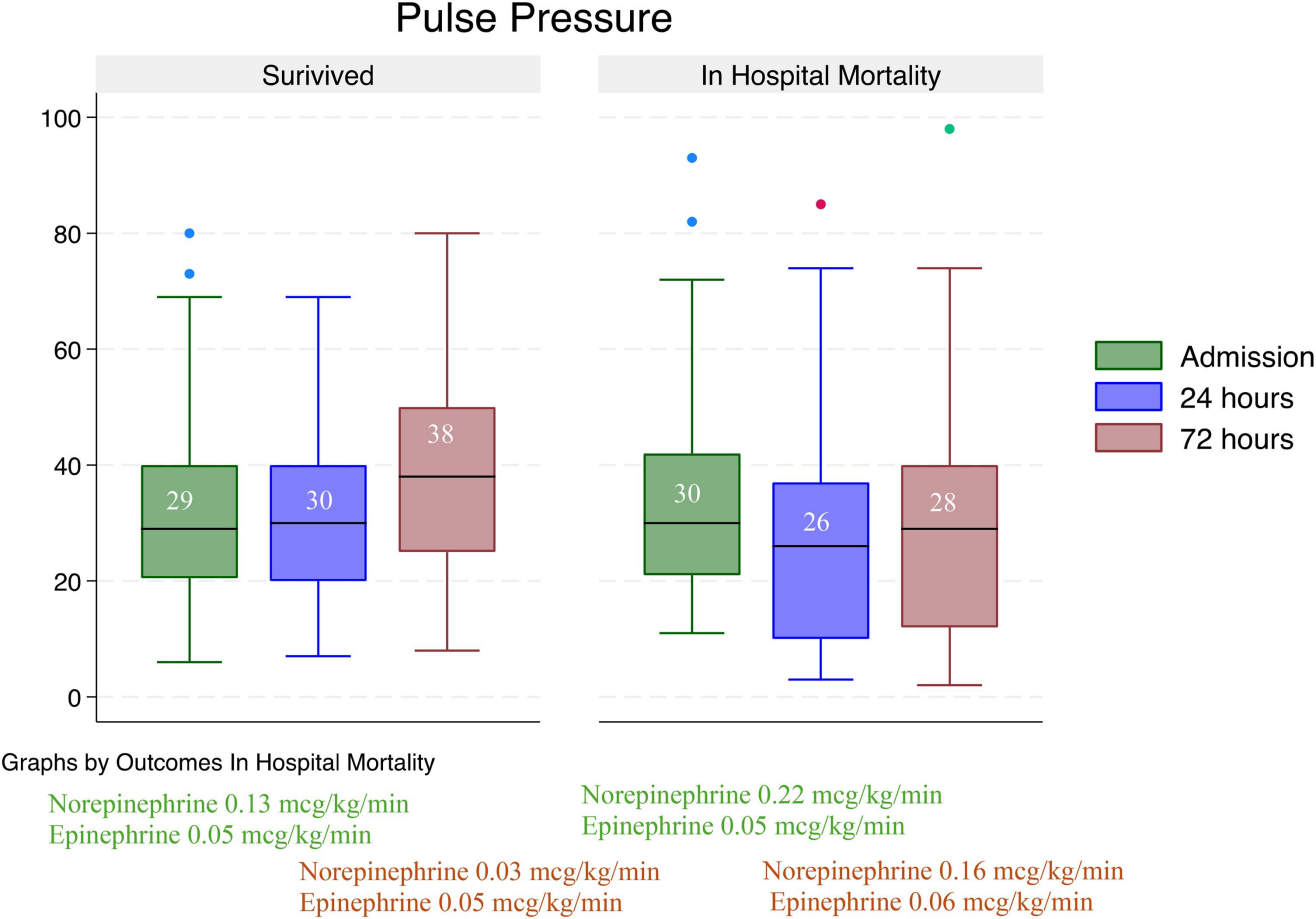
Pulse pressure during admission, 24 hours, and 72 hours post intervention and Outcomes. Among the survived group the pulse pressure showed an improvement from admission to 72 h post intervention. There was a significant reduction of norepinephrine from admission to 72 h post intervention among the survival group. The In Hospital Mortality group showed a no significant recovery by 72 h post intervention with a not a significant reduction of norepinephrine from admission to 72 h post intervention.

Serial hemodynamic assessments at admission, 24-hours and 72-hours post intervention demonstrated several differences between the survivors and the non-survivors until hospital discharge. Both survivors and non-survivors exhibited statistically significant improvements in cardiac power output during the first 24-hours post intervention. However, this early hemodynamic benefit was transient in non-survivors with cardiac power output eventually plateauing without further significant gains, while survivors continued to demonstrate significant improvements over the full 72-hour period (Figure 5). Similarly, survivors achieved significant improvement of their pulse pressure over the full 72-hours, whereas non-survivors failed to sustain their initial 24-hour improvement over the same period (Figure 6). Both observations suggest that transient early hemodynamic recovery may not be a reliable predictor of overall survival, underscoring the importance of sustained myocardial function improvement.

**Figure 7:**
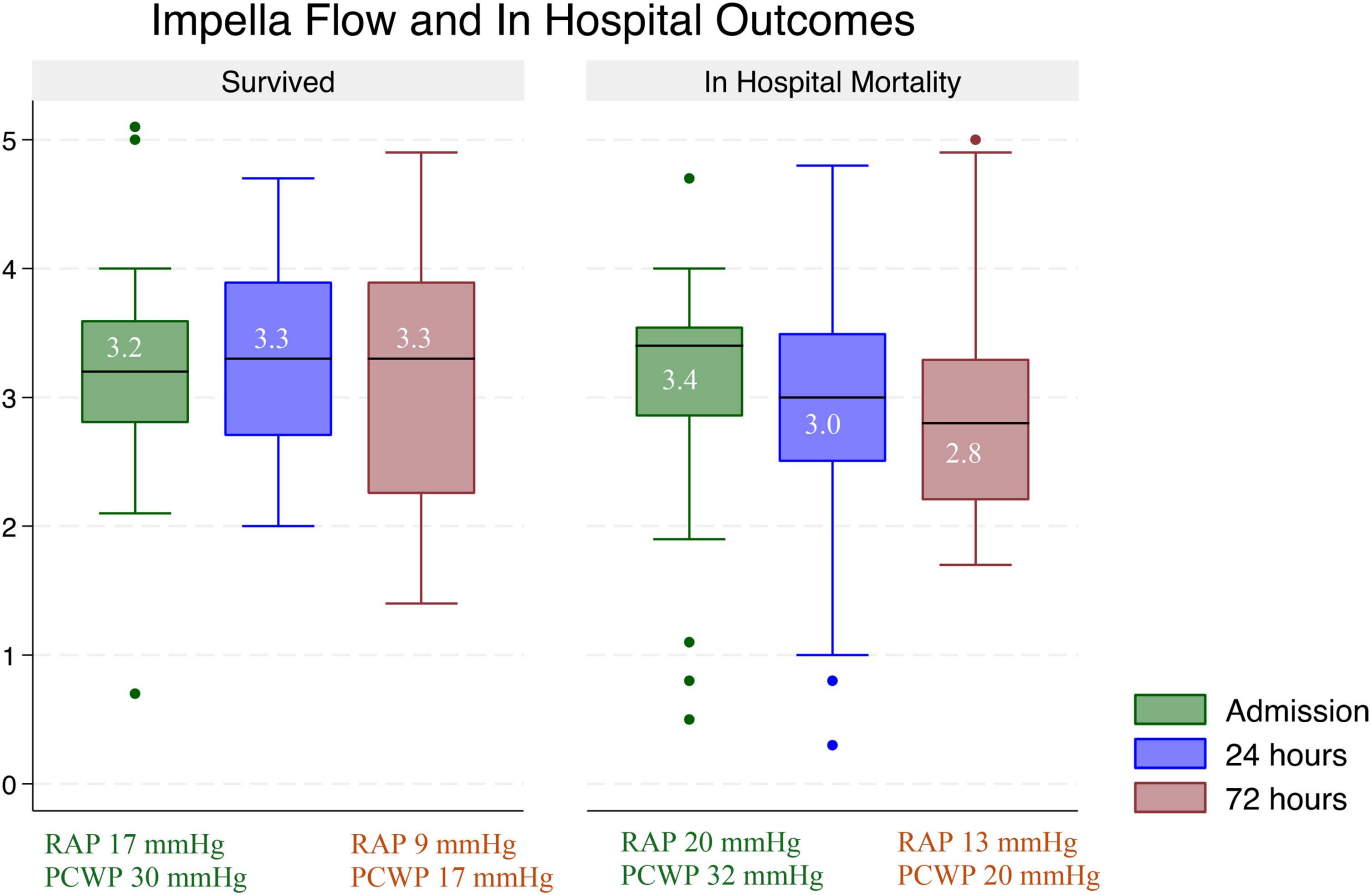
Impella flow during admission, 24 hours, and 72 hours post intervention and Outcomes. The survival group maintained the impella flow from admission all over 72 h post intervention with an improvement of RA mean pressure and PCWP mean pressure from admission to 72 h. The In Hospital Mortality group had a persistent drop of the impella Flow from admission to 72 h post intervention despite of the improvement of the RA mean pressure and PCWP mean pressure.

**Figure 8:**
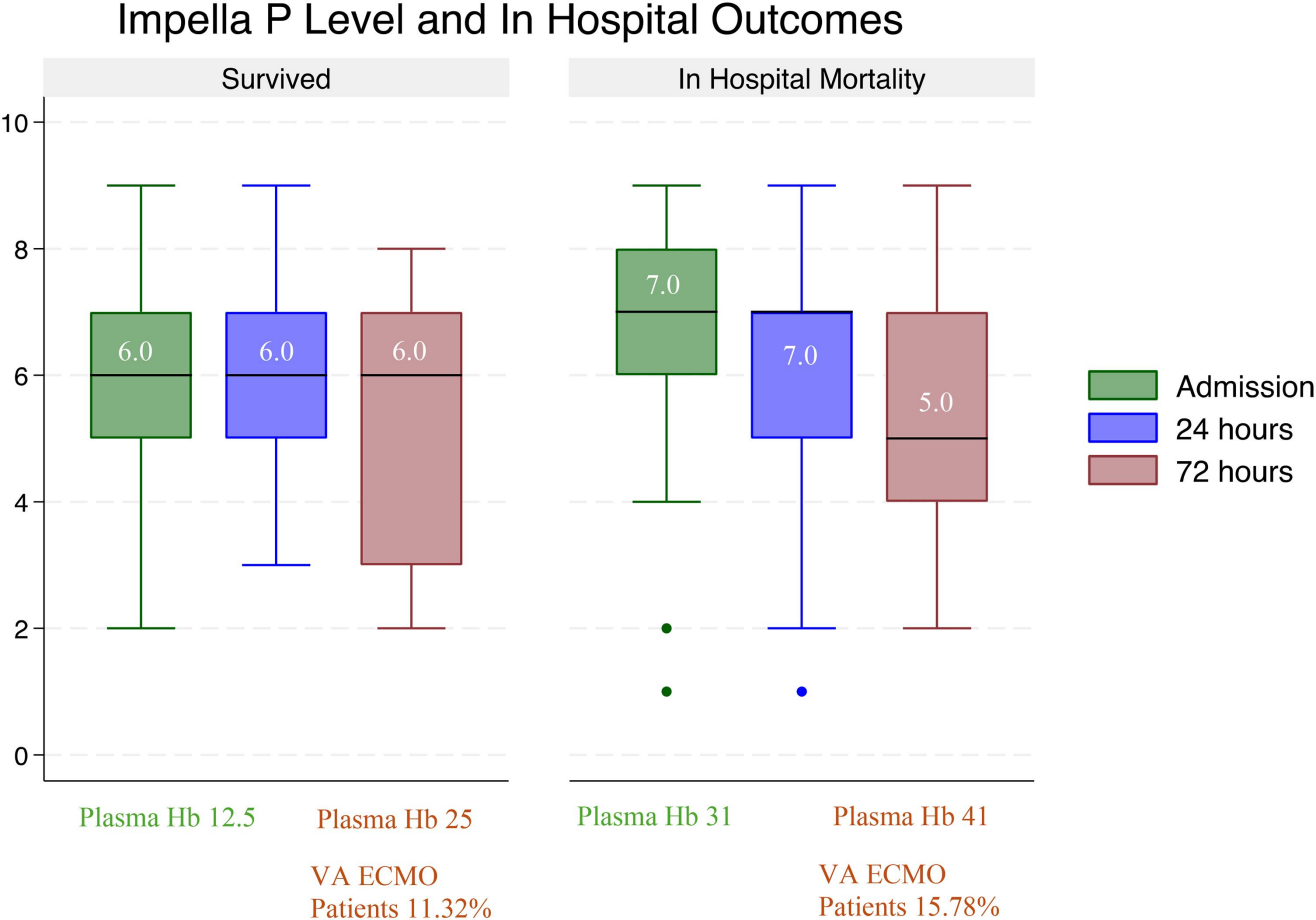
Impella P Level during admission, 24 hours, and 72 hours post intervention and Outcomes. The survival group maintained the P level from admission all over 72 h post intervention. The In Hospital Mortality group had a drop of the Impella power from admission to 72 h post intervention with persistent elevated plasma free hemoglobin.

Additionally, we observed that while the survival group maintained consistent Impella flow throughout the 72-hour period following intervention, the non-survival group showed a declining Impella flow at 72- hours. Despite these trends, both groups demonstrated significant reductions in right atrial mean pressure and pulmonary capillary wedge pressure, suggesting improving the filling pressures alone may not fully explain clinical outcomes (Figure 7). Impella power level was found stable over the 72-hour period in survivors in contrast to the in-hospital mortality group who experienced a concomitant drop in Impella power levels (Figure 8).

Lactate serum levels were significantly higher in the non-survival group at 72-hours post intervention, indicating reduced tissue perfusion and shifting of cellular metabolism towards anaerobic glycolysis. Plasma free hemoglobin levels were significantly reduced in the survivor group consistent with reduced hemolysis and more favorable MCS device – patient interaction in patients with better clinical outcomes.

Comparative analysis showed that patients who received Impella CP as the initial MCS device had a lower in-hospital mortality rate compared with those patients who were managed initially with inotropes, vasopressors and/or IABP prior to escalation. The above finding suggests that delaying advanced mechanical support may compromise patient outcomes, even when patients ultimately receive the same MCS device. Myocardial recovery was more common in the non-escalation Impella CP group when compared with the escalation group (36% vs. 24%) while heart transplantation occurred more frequently in this cohort (8% vs. 5%), indicating that early definitive support may facilitate myocardial salvage and candidacy for durable therapies.

Comparative data from prior retrospective and prospective registries contextualize our results. Results from a retrospective single center study of patients with cardiogenic shock managed with Impella CP demonstrated in-hospital mortality of 66.7% in acute myocardial infarction-related shock (AMI-CS) and 74% in those with non-AMI-CS. Among survivors 16% underwent heart transplantation and 4% received LVADs (8). In a Japanese nationwide registry, 2,047 patients with cardiogenic shock received Impella devices, 90.7% of which were Impella CP devices. In-hospital mortality rates were 46% in AMI-CS and 43.9% in non-AMI-CS patients (*p* = 0.38), with no patients receiving heart transplantation or LVAD (9).

Our study of 110 patients demonstrated 63% in hospital mortality rate in AMI-CS and 41.3% in non-AMI-CS patients reaching statistical significance (*p* = 0.027) (Figure 10). Among patients who underwent escalation to Impella 5.0/5.5, in-hospital mortality was 31%. Of the survivors, 32% had heart transplantation and 29% received LVAD, while only 8% demonstrated myocardial recovery. These findings closely mirror those reported in the Cardiogenic Shock Working Group (CSGW) multicenter registry which included 754 recipients of Impella 5.0/5.5 and reported an in-hospital mortality of 32.9%, myocardial recovery in 20.4%, heart transplantation in 23.7% and LVAD implantation in 22.1% (10). In this cohort of patients, the Impella power level remained stable over the 72-hour period, while flow demonstrated a non-significant increase. These trends are consistent with a deliberate clinical management strategy of afterload reduction and vasopressor weaning during the early phase of support.

**Figure 9:**
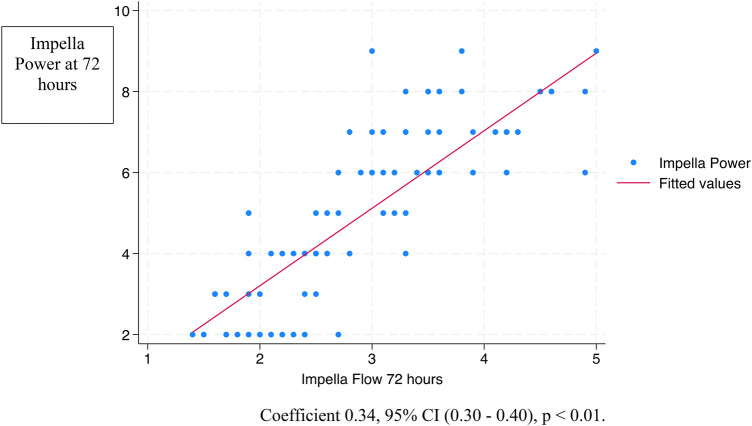
Linear correlation analysis of impella power and impella flow showed a weak positive correlation (coefficient of 0.34, 95% CI 0.30–0.40, *p* < 0.01.

**Figure 10:**
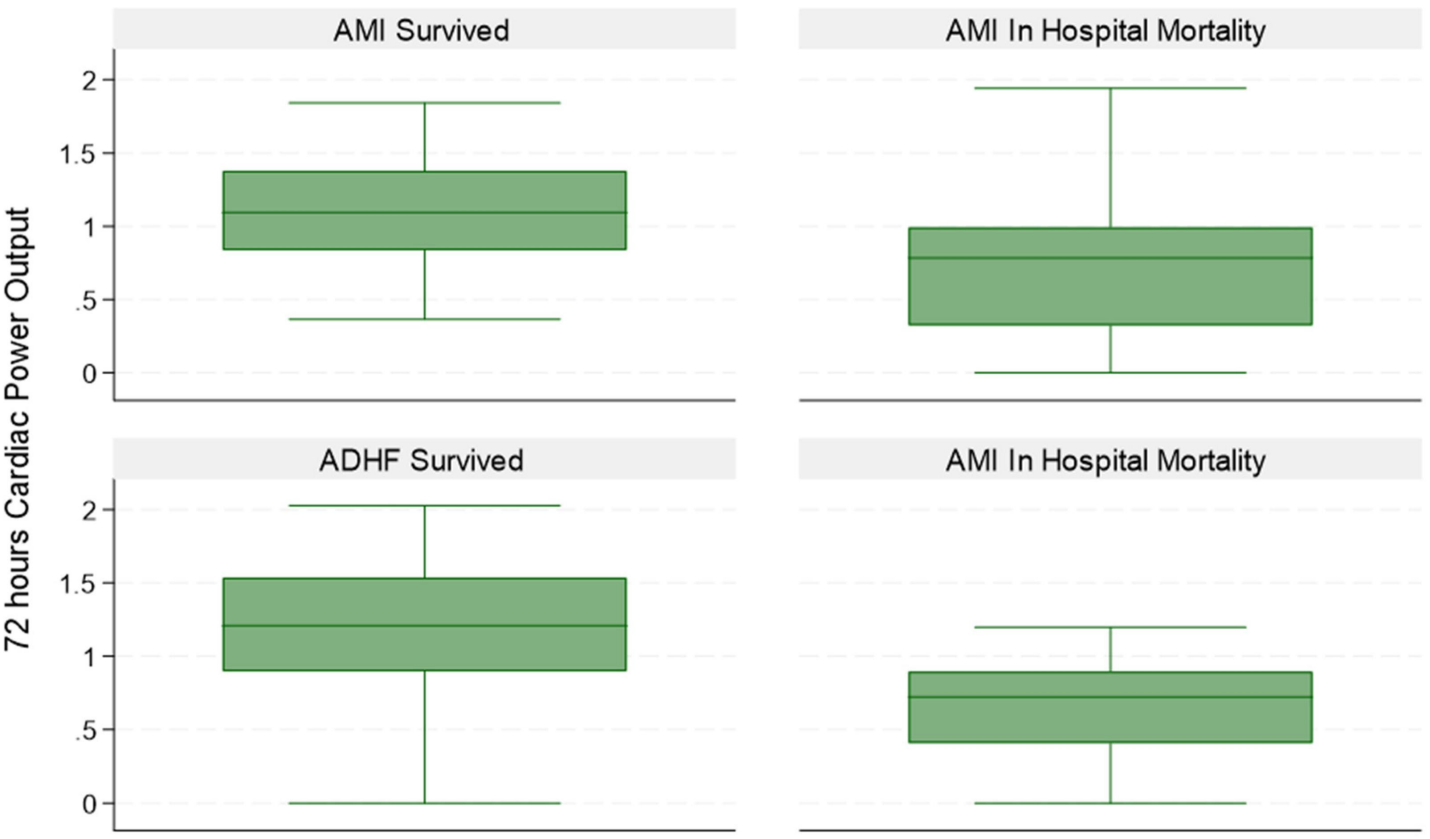
Cardiac power output by cardiogenic shock-acute myocardial infarction vs. Cardiogenic Shock-Acute Decompensated Heart Failure and Outcomes. The average of the cardiac power output (CPO) by 72 h post intervention showed that the survival group had CPO > 1.0 regardless of the etiology (CS-ADHF vs. CS-AMI) compared with In-Hospital Mortality groups that had CPO < 1.0.

**Figure 11:**
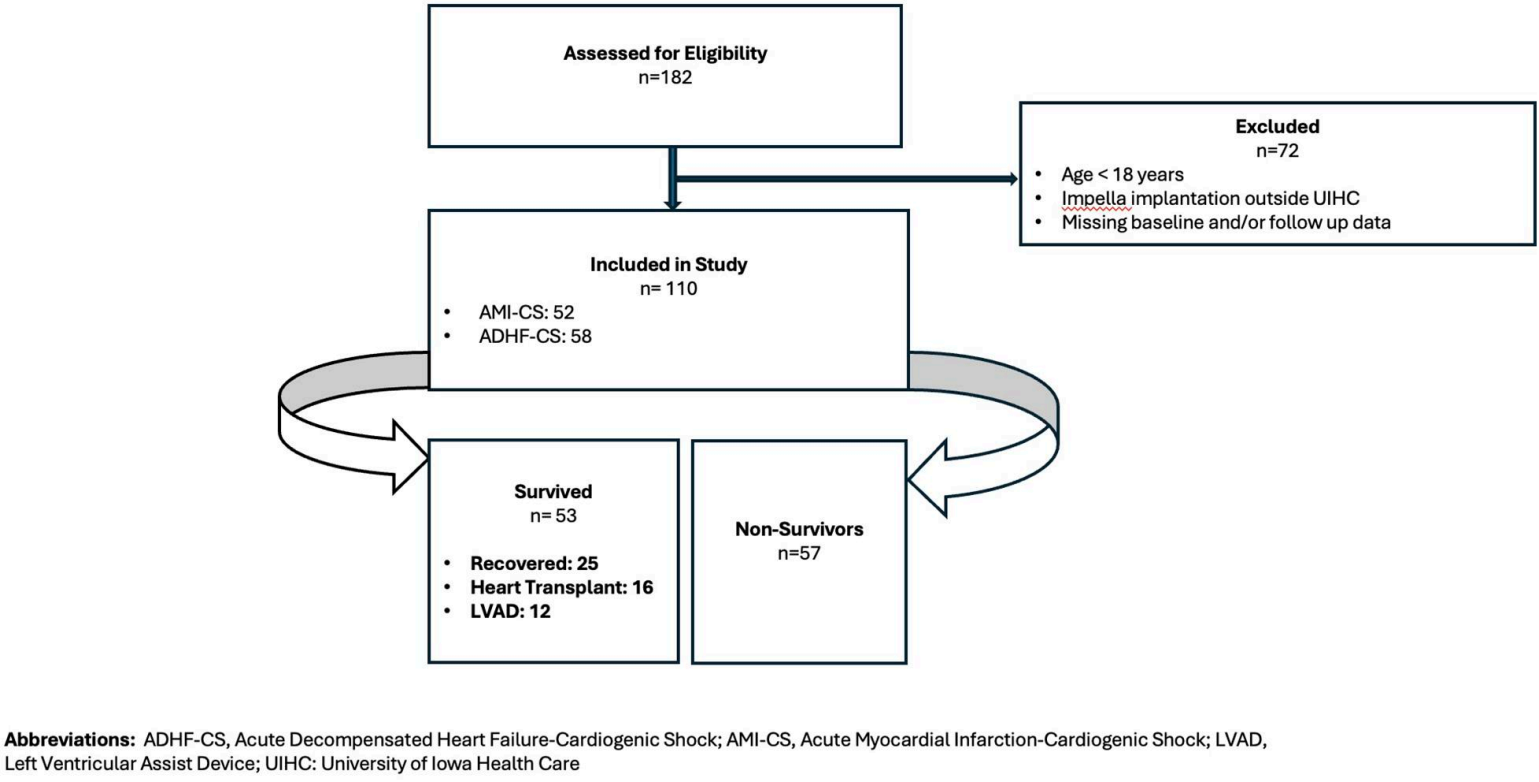
Patient eligibility and clinical outcomes flowchart. Among 182 patients assessed for mechanical circulatory support, 72 were excluded and 110 were included in the final analysis. The included cohort consisted of 52 patients with acute myocardial infarction–cardiogenic shock (AMI-CS) and 58 patients with acute decompensated heart failure–cardiogenic shock (ADHF-CS). Overall, 53 patients survived to hospital discharge, including 25 with myocardial recovery, 16 who underwent heart transplantation, and 12 who received LVAD placement; 57 patients did not survive to hospital discharge.

Among patients who underwent escalation to combined VA-ECMO and Impella support, in-hospital mortality was 90% with myocardial recovery observed in only 10%. In comparison, in a single center analysis of 44 patients with cardiogenic shock treated with VA-ECMO and Impella reported an in-hospital mortality rate of 40.9% with 36.4% achieving myocardial recovery and 22.8% undergoing either heart transplantation or LVAD implantation (11). Differences may reflect variation in patient selection, timing of escalation or center specific practices. In our population, Impella power levels increased over the 72-hour period accompanied by a significant increase in flow. These trends may reflect a targeted strategy to unload the left ventricle by reducing ECMO flow and vasopressor requirements during hemodynamic stabilization.

The de-escalation strategy from VA-ECMO to Impella 5.5 lead to 100% of patients surviving hospital discharge, with 40% of patients successfully bridged to heart transplantation and 20% to LVAD placement. Myocardial recovery occurred in 20% of cases. This approach remains poorly characterized in the literature, as no large registries or retrospective analyses have systematically evaluated outcomes of this de-escalation approach. Hemodynamically, these patients demonstrated increasing Impella power levels over the 72-hour period, alongside with a significant improvement of the Impella flow, consistent with clinical intent of weaning of ECMO support and vasopressors to promote left ventricular unloading.

### Limitations

This single-center study requires external validation in larger, multicenter cohorts. The events-per-variable ratio limited multivariable model complexity; models with more than two covariates should be interpreted as exploratory. Although pulmonary hemodynamic data were available, comprehensive hemodynamic assessment of right ventricular function was not performed. Formal intra- and inter-observer reproducibility analysis with quantitative agreement metrics was not performed. Treatment heterogeneity was not accounted for in the survival analysis.

## Conclusions

Overall, our data indicate that sustained hemodynamic improvement, particularly in cardiac power output and pulse pressure, is associated with survival, independently of absolute changes in Impella flow (Figure 9) (12). Early initiation of MCS, preservation of myocardial performance and titration of afterload and vasopressors appear central to optimizing outcomes in cardiogenic shock.

Escalation strategies involving Impella 5.0/5.5 or VA-ECMO may benefit select patients, although high mortality in VA-ECMO recipients underscores the importance of careful patient-candidate selection (13). De-escalation from VA-ECMO to Impella 5.5 showed promising outcomes, suggesting potential utility as a bridge-to-recovery or definitive therapy. Future studies should potentially focus on optimal timing and sequencing of MCS modalities, tailoring them to individual hemodynamic trajectories. Additionally, further exploration of the de-escalation strategy to Impella 5.5 is warranted given its apparent survival benefit in our cohort.

## Data Availability

The raw data supporting the conclusions of this article will be made available by the authors, without undue reservation.
